# Protective Effect of Anthocyanins from Lingonberry on Radiation-induced Damages 

**DOI:** 10.3390/ijerph9124732

**Published:** 2012-12-18

**Authors:** Zi-Luan Fan, Zhen-Yu Wang, Li-Li Zuo, Shuang-Qi Tian

**Affiliations:** 1 School of Forestry, Northeast Forestry University, 26 HeXing Road, XiangFang District, Harbin 150040, China; E-Mail: fzl_1122@163.com; 2 School of Food Science and Engineering, Harbin Institute of Technology, 73 HuangHe Road, NanGang District, Harbin 150090, China; E-Mail: zuolili213@163.com; 3 College of Food Science and Technology, Henan University of Technology, Zhengzhou 450001, China; E-Mail: tianshuangqi2002@163.com

**Keywords:** lingonberry, anthocyanins, radioprotection, immunomodulatory

## Abstract

There is a growing concern about the serious harm of radioactive materials, which are widely used in energy production, scientific research, medicine, industry and other areas. In recent years, owing to the great side effects of anti-radiation drugs, research on the radiation protectants has gradually expanded from the previous chemicals to the use of natural anti-radiation drugs and functional foods. Some reports have confirmed that anthocyanins are good antioxidants, which can effectively eliminate free radicals, but studies on the immunoregulatory and anti-radiation effects of anthocyanins from lingonberry (ALB) are less reported. In this experiment, mice were given orally once daily for 14 consecutive days before exposure to 6 Gy of gamma-radiation and were sacrificed on the 7th day post-irradiation. The results showed that the selected dose of extract did not lead to acute toxicity in mice; while groups given anthocyanins orally were significantly better than radiation control group according to blood analysis; pretreatment of anthocyanins significantly (*p* < 0.05) enhanced the thymus and spleen indices and spleen cell survival compared to the irradiation control group. Pretreatment with anthocyanins before irradiation significantly reduced the numbers of micronuclei (MN) in bone marrow polychromatic erythrocytes (PCEs). These findings indicate that anthocyanins have immunostimulatory potential against immunosuppression induced by the radiation.

## 1. Introduction

Radiation, oxidation, and toxic substances are all potential damaging factors, which are associated with environmental pollution and immunity to disease. Radiation is one of the most important environmental factors and has hazardous effects on health, which include oxidative stress [1,2], hematopoietic system dysfunction [3], genetic mutations [4] and immune dysfunction [5]. People may be exposed to ionizing radiation during radiotherapy which generates free radicals when it passes through living tissues. Interactions of free radicals with DNA can induce genetic damage, leading to mutagenesis and carcinogenesis [6]. During early development of effective radiomodifiers, some have focused on synthetic thiol compounds, such as amifostine [7]. Due to its side effects and toxicity, the use of this drug is limited in clinical practice, though it reduces mortality. In recent years, research on radiation protectants has gradually expanded from the previous synthetic compounds to the search for natural anti-radiation drugs and functional foods. Thus, the development of less toxic natural compounds could be of great interest. 

Phenolic compounds are a wide group of aromatic compounds that exist naturally in plants and berries [8], including, for example, flavonoids and aromatic acids produced via the shikimate and acetate pathways in plants. They have a wide array of biological properties, including antibacterial, antiviral, anticancer [9], immunostimulant and antioxidant effects [10]. Lingonberry (*Vaccinium vitis-idaea *L.) is a wild, semi-woody chamephyte that keeps its leaves through winter and commonly grows in northern latitudes [11]. Lingonberry is the most abundantly picked wild berry in the Lesser Khingan and Greater Higgnan Mountains area of Heilongjiang Province, China. The most abundant phenolic compounds in lingonberries are anthocyanin glycosides, natural pigments responsible for most blue and red colors in berries [12]. In the past few years lingonberry products, together with another phytochemically similar berry from the *Vaccinium *genus, cranberry, have been increasingly marketed as a natural solution for the treatment of urinary tract infections [13]. Some reports have confirmed that anthocyanins are good antioxidants, which can effectively eliminate free radicals, but information about the immunoregulatory and anti-radiation effects of anthocyanins from lingonberry *in vivo *is limited. In this study, we investigated the possibility that anthocyanins extracted from the fruits of lingonberry (ALB) would be able to protect the body from radiation injury by improving immune function and reducing DNA damage, thus reducing the risk of developing radiation-related diseases. With the results of the study, ALB may become a more available new drug to accord with international medicine standardization and have better therapeutic value in clinic than existing products. 

## 2. Materials and Methods

### 2.1. Plant Material

Lingonberries were harvested at the light ripe red stage of ripening from the Greater Higgnan Mountains area of Heilongjiang Province, China. The fresh fruits were frozen and then stored at −40 °C prior until further analysis.

### 2.2. Preparation of Anthocyanins from Lingonberry

Fresh fruits were homogenised with chilled 80% acetone (1:2; w/v) using a chilled JJ-2 Tissue disintegrator for 5 min. The sample was then further homogenised using a FA25 Superfine Homogeniser for an additional 3 min [14]. The homogenates were filtered and evaporated. The solvents were evaporated to dryness under vacuum and the residue was diluted in distilled water. After separating the precipitate by filtration; the remaining solution was further separated on an adsorptive resin column (X-5; Nankai University, Tianjin, China) [15] and eluted with water, 20%, 30%, 40%, 50%, 60%, 80% and 95% EtOH to produce eight fractions. The 30%-EtOH fraction was dried under vacuum, recrystallized from EtOH, and a red powder was obtained containing the ALB. 

### 2.3. Animals

Male and female Kunming mice (24 ± 2 g) of clean grade were provided by the Harbin Medical University Animal Center (SCXK20020002, Harbin, China). The animals were conditioned for a week at 23 ± 1 °C with a constant humidity of 55% ± 5% under a cycle of 12 h of dark [16], and had free access to food and tap water according to GLP. All experimental protocols used in this experiment have previously been approved by the School of Medical Science, Harbin Medical University. 

A total of 60 mice were used for these experiments, with 10 mice per group. Mice were randomly assigned to one of the six following treatment groups: normal control, model, positive control (Leucogen 1.4 mg/kg body weight/day) and ALB-H(igh), M(edium), L(ow) dose (200, 100, 50 mg/kg body weight/day). Different doses of ALB dissolved in double distilled water were administered intragastrically to the female and male mice for 14 consecutive days. The animals were divided into six groups: Positive group was administered Leucogen tablets (Ji Bei er Medicine Co. Ltd., Jiangsu, China, 10 mg/pellet, batch number: 110304). Model and normal groups were orally administered with distilled water. All mice, except the Normal control group, were exposed to 6.0 Gy ^60^Co γ-ray whole-body radiation on day 15 after administration of DDW or ALB. The animals were monitored daily for the development of symptoms of radiation sickness and mortality.

### 2.4. Irradiation

The radiation centre of the Heilongjiang Academy of Agricultural Sciences was used for the irradiation experiments. Mice were restrained in well-ventilated boxes and exposed to whole-body γ-radiation (6 Gy), at a dose rate of 1.01 Gy/min at a source-to-animal distance (midpoint) of 160 cm. 

### 2.5. Blood Cell Count

Blood cell count (leucocytes-WBC, erythrocytes-RBC, hemoglobin-HGB and thrombocytes-PLT) was determined by use of hemocounter (Haematology Analyser 6318K, Nihon-Konden, Tokyo, Japan) on the 7th day after irradiation. For this purpose blood samples were taken from the eyeballs of animals. 

### 2.6. Thymus and Spleen Indices

Animals were given ALB (50, 100, 200 mg/kg b.wt.) prior to 6.0 Gy whole-body irradiation. On day 7 post-irradiation, animals were sacrificed and the spleen and thymus were removed. The spleen and thymus indices were calculated by dividing organ weight by BW [17]:

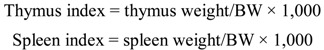



### 2.7. Measurement of Carbon Clearance and Phagocytic Index (PI)

At day 7 after radiation the mice were injected intravenously via a lateral tail vein with India ink stain (Beijing Zhongxi Chemical Factory, Beijing, China) at a dose of 0.1 mL/10 g body weight (1:4 dilluted with PBS). Blood samples (20 μL of each) were taken from the retroorbital venous plexus at 2 min (t_1_) and 10 min (t_2_) intervals respectively after India ink staining injection and mixed with 2 mL 0.1% Na_2_CO_3_ in tube for measuring the optical density with spectrophotography at 600 nm (OD_1_ for 2 min and OD_2_ for 10 min) [18]. The PI was calculated according to:

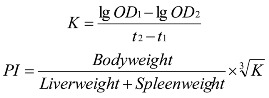



### 2.8. Splenocyte Proliferation Index

Sterile spleens were obtained at day 21. The spleen was pulverized through a 200-mesh filter and washed with 5 mL phosphate-buffered saline (PBS). The spleen cell suspension was washed three times in PBS in RPMI-1640 culture medium with 10% fetal calf serum and the cell concentration was adjusted to 2 × 10^6^/mL in 96-well plates with 90 μL/well. Experiments were repeated three times. Concanavalin A (Con A, 15 μg/well) was added to the wells. Culture medium was added to negative control wells in place of Con A. The cells were cultivated in 5% CO_2_ at 37 °C for 72 h and 5 mg/mL methylthiazolyl tetrazolium (MTT) salt was added after 4 h. The cell suspension was acidified with 10% sodium dodecyl sulfate after 72 h to enable the purple crystals to completely dissolve. The optical density (OD) was determined at 570 nm [5]. The proliferation index = (value for experimental well − value for the control well/value for control well) × 100%.

### 2.9. Effect of ALB on Bone Marrow Micronuclei Formation

Femur heads were obtained from mice at day 21. They were washed with 5 mL PBS and centrifuged at 1,000 rpm for 10 min. The supernatant was discarded and the sediment was applied to a slide covered with serum. The smear was fixed in methanol solution for 10 min and then stained with Giemsa for 10–15 min. The presence of micronuclei was observed in five evenly-dispersed visual fields under a high-power microscope. Bone marrow polychromatic erythrocytes appeared blue and mature red blood cells appeared orange. Most micronuclei were round or oval-shaped, single or multiple, and their edges were smooth and regular. The micronuclei rates (‰) were calculated with the number of micronuclei in 1,000 polychromatic erythrocytes [19].

### 2.10. Statistical Analysis

All of the experimental data at least in triplicate are expressed as the means ± SD. Statistical significance was evaluated using the two-tailed, unpaired Student’s t-test for comparisons between two means. A value of *p *< 0.05 was considered to indicate statistical significance.

## 3. Results

### 3.1. Effect of ALB on Body Weight and Peripheral Blood Counts of Irradiated Mice

**Figure 1:**
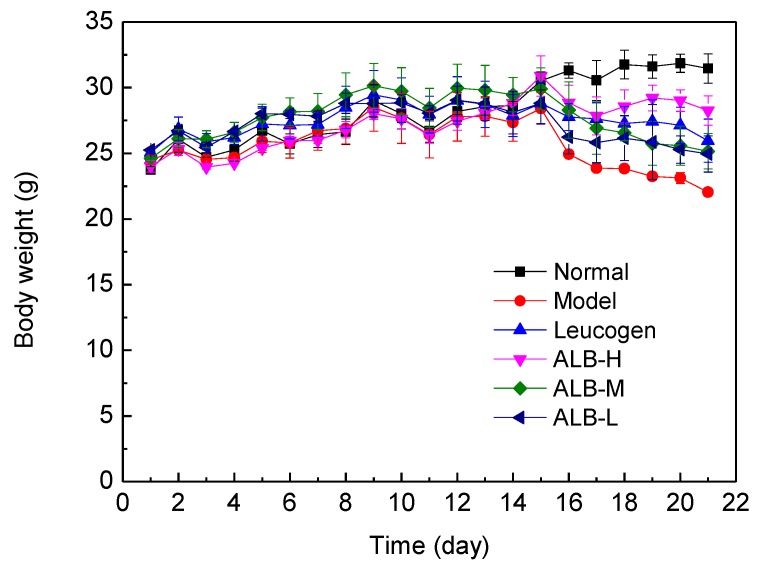
The changes of body weight of mice caused by irradiation. The ALB-H/M/L groups were given ALB daily by intragastric administration based on dose of 200, 100, and 50 mg/kg body weight (b.w.) per day for 14 consecutive days. All mice, except the Normal control group, were exposed to 6.0 Gy ^60^Co γ-ray whole-body radiation on day 15.

ALB administration was not associated with any mortality throughout the whole observation period. Figure 1 shows the growth curve of body weight in mice. The average weight of mice in each group at the 14th days (before irradiation) compared with the beginning of weight was increased. However, the rate of increased body weight between the groups was no significant difference; this proves that the selected dose of administration group and positive control group in mice has no acute toxicity [20]. 

Seven days after irradiation, the body weight of the control group (no radiation) continued to increase, the other five groups showed weight decreases compared with the previous body weight. The radiation model group showed varying degrees of hair loss, reduction in activity, food and water intake decreased, the tail roots appeared stained and so on. Compared with the radiation model, ALB-H group and the positive control group showed reduced rates of weight loss. These results indicated that ALB-H and Leucogen can alleviate the weight loss due to the radiation. ALB-treated irradiated groups has not shown any significant dose-dependency.

The variations of the mean values for WBC, RBC, PLT and HGB parameters after exposure are shown in Table 1, which demonstrated a significant (*p* < 0.01) decrease for the model group exposed to 6 Gy radiation, compared with the unirradiated control group. A significant (*p* < 0.01) increase in the total WBC, RBC, PLT and HGB count compared to the model group was recorded for the Leucogen and ALB groups; among of them the in crease of RBC was the most highest, and was insignificantly different from the unirradiated control group (*p *> 0.05). Increase of WBC counts was not high, especially in the ALB-M and ALB-L groups (*p* > 0.05). The peripheral blood counts in the Leucogen group were similar to those of the ALB-M group (Table 1). These results suggest that ALB could reduce the effects of radiation on hemograms in mice.

**Table 1:** The effects of lingonberry anthocyanins on blood cell analysis of radiation in mice (mean ± S.D, n = 10) (^**^ p < 0.01, ^*^ p < 0.05 *vs.* Normal; ^▲▲^* p *< 0.01, ^▲^* p *< 0.05 *vs.* Model).

Group	WBC (10^9^/L)	RBC (10^12^/L)	HGB (G/L)	PLT (10^9^/L)
Normal	5.81 ± 0.66	9.62 ± 1.48	141.14 ± 14.7	811.71 ± 106.84
Model	0.45 ± 0.26^**^	3.54 ± 0.92^**^	28.14 ± 3.33^**^	63.29 ± 19.46^**^
Leucogen (1.4 mg/kg)	0.81 ± 0.26^**▲^	9.17 ± 1.40^▲▲^	39.29 ± 9.79^**▲^	208.14 ± 83.14^**▲▲^
ALB-H (200 mg/kg)	0.71 ± 0.12^**▲^	9.28 ± 1.10^*▲▲^	102.14 ± 7.76^**▲▲^	219.43 ± 73.89^**▲▲^
ALB-M (100 mg/kg)	0.59 ± 0.18^**^	7.64 ± 1.11^▲▲^	67.43 ± 6.50^**▲▲^	286.86 ± 85.55^**▲▲^
ALB-L (50 mg/kg)	0.61 ± 0.17^**^	8.07 ± 0.91^*▲▲^	62.71 ± 9.50^**▲▲^	127.57 ± 58.55^**▲^

Hematopoietic tissues and the immune system are highly sensitive to radiation. Radiation damage to the hematopoietic system affects the hematopoietic stem cells and reduces their cell proliferation capacity [21]. Inhibition of hematopoiesis is one of the clinical symptoms of radiation injury. Radiation injury induced by radiation is usually associated with immune system disease and a decline in white and red blood cells [22]. The results of the current study found that WBC, RBC, PLT, and total HGB in the peripheral blood of irradiated mice were increased by ALB, thus improving hematopoietic function and survival.

### 3.2. Effect of ALB on Immune Function

Total-body irradiation of mice with 6 Gy 60Co γ-rays significantly reduced thymus and spleen indices (*p* < 0.01). The spleen indices were significantly higher in all ALB-treated irradiated groups, in a dose-dependent manner, compared with the model group (*p *< 0.05, *p* < 0.01). The thymus indexes in the ALB 200 mg/kg and 100 mg/kg groups were significantly higher than that in the irradiated control group (*p* < 0.01) (Table 2). These results suggest that ALB exerted a protective effect by increasing immune organ indices. 

**Table 2:** The effects of lingonberry anthocyanins on organ/body weight ratio, phagocytic capacity of radiation in mice (mean ± S.D, n = 10) (^**^ p < 0.01, ^*^ p < 0.05 *vs.* Normal; ^▲▲^* p* < 0.01, ^▲^* p *< 0.05 *vs.* Model).

Group	n	Spleen index	Thymus index	Phagocytic capacity
Normal	10	4.24 ± 0.17	3.06 ± 0.17	7.33 ± 0.25
Model	10	2.02 ± 0.11^**^	1.22 ± 0.03^**^	5.92 ± 0.38^**^
Leucogen (1.4 mg/kg)	10	2.16 ± 0.18^**^	1.49 ± 0.18^**▲^	6.57 ± 0.34^*▲^
ALB-H (200 mg/kg)	10	3.01 ± 0.22^**▲▲^	2.01 ± 0.23^**▲▲^	6.68 ± 0.32^*▲^
ALB-M (100 mg/kg)	10	2.27 ± 0.19^**▲^	1.45 ± 0.09^**▲▲^	7.03 ± 0.51^▲^
ALB-L (50 mg/kg)	10	2.23 ± 0.17^**▲^	1.39 ± 0.18^**^	7.02 ± 0.39^▲▲^

### 3.3. Effect of ALB on Phagocytotic Function of Mononuclear Phagocytic System (MPS)

The PI value obtained in mice pretreated differently is shown in Table 2. Significant decrease in the rate of carbon clearance in irradiated mice was observed compared with normal control (*p* < 0.01). Combined with ALB preteatment, there were no significant differences (*p* > 0.05) in the decrease in the rate of carbon clearance induced by radiation. ALB 50, 100, 200 mg/kg revealed more strongly an accentuation of the phagotrophy function. The rate of carbon clearance in groups treated with ALB was elevated compared with the model group (*p* < 0.05, *p* < 0.01), among them, the group with ALB 50 mg/kg showed the greatest PI value.

### 3.4. Effect of ALB on Splenocyte Proliferation

**Figure 2:**
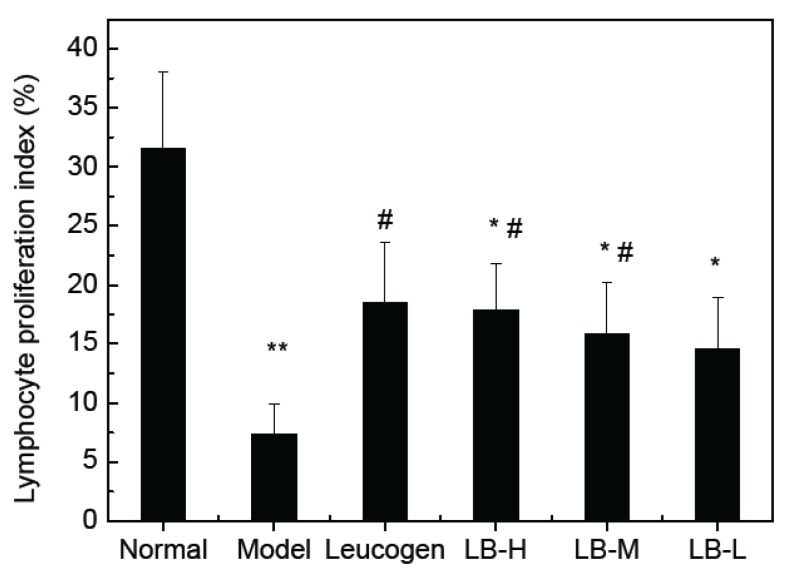
The effects of lingonberry anthocyanins on spleen cell proliferation of radiation in mice (mean ± S.D, n = 10) (^**^ p < 0.01, ^*^ p < 0.05 *vs.* Normal; ^#^* p *< 0.05 *vs.* Model).

To understand the immunomodulatory activity of ALB from lingonberry, we investigated its effects on the proliferation of spleenic cells. The irradiated mice groups were obviously found to display significantly decreased proliferation of splenocytes compared with that of normal control (*p* < 0.05, *p* < 0.01). All of the ALB test doses (50–200 mg/kg) significantly increased the proliferation of spleenic cells in a dose dependent manner compared with the model group, but less than positive control group (Figure 2). Con-A (5 μg/mL) stimulated spleenocyte proliferation was significantly enhanced by Leucogen at 1.4 mg/kg and cellular proliferation was increased up to three fold in Con-A treated cells, compared to the model group.

### 3.5. Effect of ALB on Bone Marrow Micronucleus Formation in Mice

**Figure 3:**
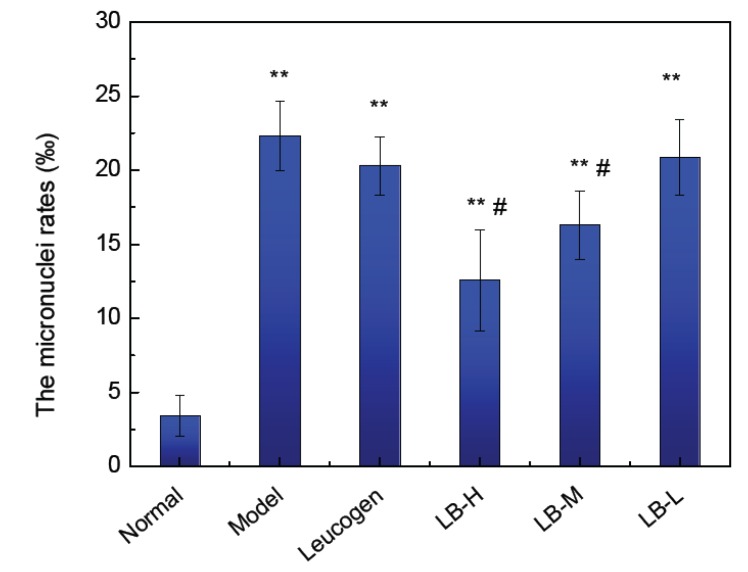
Effect of lingonberry anthocyanins on the development of micronuclei in bone marrow (^**^ p < 0.01, ^*^ p < 0.05 *vs.* Normal; ^#^* p *< 0.05 *vs.* Model).

The incidence of micronuclei (Mn) in the bone marrow was significantly increased by irradiation (*p* < 0.01). The bone marrow micronuclei rates in the 200 mg/kg ALB, 100 mg/kg ALB and were significantly lower than in the model group, (*p* < 0.05). The bone marrow micronuclei rate in the 50 mg/kg ALB group was similar to that in the positive groups with Leucogen (Figure 3). These results suggest that high doses of ALB inhibited the production of radiation-induced micronuclei. 

Micronuclei formation, which assesses the degree of DSBs in DNA, is one of the best assays for the evaluation of genome instability induced by ionizing radiation [23]. This method is performed *in vitro* (e.g., in human cultured lymphocytes) and *in vivo* (e.g., in mouse bone marrow cells and peripheral blood leucocytes). Higher genome instability results in a higher frequency of Mn. Pretreatment of human culture lymphocytes with ALB statistically significant reduced the Mn frequencies that were increased by IR. These ALB did not increase the frequency of Mn in non-irradiated human lymphocyte cultures treated at IR protective doses. The bone marrow is damaged at the molecular level by radiation, which causes chromosome aberrations and increased development of micronuclei [24,25]. Micronucleus detection can therefore be used as a diagnostic biological indicator of radiation damage, accurately reflecting the degree of chromosome damage and repair capacity. The bone marrow micronuclei rates detected in the current study showed that ALB significantly reduced DNA damage.

## 4. Discussion

Humans have always been exposed to natural background radiation. Reactive oxygen species (ROS) levels can be elevated by exposure to oxidative stress such as ionizing radiation (IR; e.g., X-ray, gamma ray, β, α or proton) or by deficiencies in the cellular repair process. During oxidative stress and exposure to radiation, excessive free radicals are produced [26]. This can damage crucial macromolecules including DNA, cell membranes and enzymes, and can cause cell death. DNA damage includes genotoxicity, chromosomal abnormalities, gene mutations and cell death if the damage is beyond repair [27]. The inherent antioxidant defence systems of the cell competitively counteract the oxidative stress. However, free radical insult surpassing the inherent cellular defence mechanism warrants external antioxidant supplementation. Naturally occurring antioxidants may also provide an extended window of protection against irradiation, and may have therapeutic potential when administered after irradiation. Several plant constituents have been proven to possess considerable free radical scavenging or antioxidant activity [1,28].

Fruits contain numerous bioactive components and are especially rich in phenolic compounds such as flavonoids, phenolic acids, stilbenes, and procyanidins [8]. These compounds are believed to work synergistically to promote human health through a variety of different mechanisms, such as enhancing antioxidant activity, impacting cellular processes associated with apoptosis, platelet aggregation, blood vessel dilation, and enzyme activities associated with carcinogen activation and detoxification [29,30]. Elucidating the full potential of the health promoting capabilities of fruits continues to enhance and advance the discipline of functional foods and nutraceutical research. In the present study, we demonstrated that ALB dramatically increased the survival rate of irradiated mice and had a protective effect on hematopoietic tissues and the immune system. Moreover, our results also showed that ALB could protect cells from DNA damage.

Radiation often inhibits the immune system through bone marrow suppression, reduction in the number of immune cells, and microcirculatory disturbances. Our results demonstrated that ALB protected the thymus and spleen and increased lymphocyte proliferation stimulated by Con A, suggesting that ALB could improve immunity. These results are consistent with other reports [31].

It is clear that anthocyanins reduce cell death owing to their antioxidant effects and protective effects on biomolecules such as DNA. In animal studies, administration of several flavonoids such as morin and genistein [32] statistically significant reduced mortality rates induced by IR. These effects are thought to be related to the protective effects of flavonoids and maintenance or recovery of levels of crucial cell populations such as platelets, white blood cells and hematopoietic progenitor cells, which are reduced by IR.

Much of the attention given to anthocyanins compounds comes from the results of epidemiological studies that suggest high fruit and vegetable consumption is associated with a decreased risk of several types of cancer including breast, colon, lung, larynx, pancreas, oral and prostate. These suggested protective effects of anthocyanins, together with their potent anti-oxidative and free radical scavenging activities observed in *in vitro* studies [33] have increased the public’s interest in the use of anthocyanins for their potential health benefits. Therefore, understanding the potential benefits or adverse effects of natural products that are extensively used by human population is very important to implement public health safety measures [34]. 
